# The Neutrophil/Lymphocyte Ratio at Diagnosis Is Significantly Associated with Survival in Metastatic Pancreatic Cancer Patients

**DOI:** 10.3390/ijms18040730

**Published:** 2017-03-29

**Authors:** Matteo Piciucchi, Serena Stigliano, Livia Archibugi, Giulia Zerboni, Marianna Signoretti, Viola Barucca, Roberto Valente, Gianfranco Delle Fave, Gabriele Capurso

**Affiliations:** Digestive and Liver Disease Unit, Sant’Andrea Hospital, Faculty of Medicine and Psychology, “Sapienza” University of Rome, 00189 Rome, Italy; matteopiciucchi@libero.it (M.P.); seri_stigliano@yahoo.it (S.S.); livia.archibugi@hotmail.it (L.A.); giulia.zerboni@gmail.com (G.Z.); mariannasignoretti@gmail.com (M.S.); viola.barucca@gmail.com (V.B.); robbie.valente@gmail.com (R.V.); gianfranco.dellefave@gmail.com (G.D.F.)

**Keywords:** pancreatic cancer, neutrophil/lymphocyte ratio, prognosis, biomarkers, chemotherapy

## Abstract

Different inflammation-based scores such as the neutrophil/lymphocyte ratio (NLR), the Odonera Prognostic Nutritional Index (PNI), the Glasgow Prognostic Score, the platelet/lymphocyte ratio, and the C-reactive protein/albumin ratio have been found to be significantly associated with pancreatic cancer (PDAC) prognosis. However, most studies have investigated patients undergoing surgery, and few of them have compared these scores. We aimed at evaluating the association between inflammatory-based scores and PDAC prognosis. In a single center cohort study, inflammatory-based scores were assessed at diagnosis and their prognostic relevance as well as that of clinic-pathological variables were evaluated through multiple logistic regression and survival probability analysis. In 206 patients, age, male sex, tumor size, presence of distant metastasis, access to chemotherapy, and an NLR > 5 but not other scores were associated with overall survival (OS) at multivariate analysis. Patients with an NLR < 5 had a median survival of 12 months compared to 4 months in those with an NLR > 5. In the 81 patients with distant metastasis at diagnosis, an NLR > 5 resulted in the only variable significantly associated with survival. Among patients with metastatic disease who received chemotherapy, the median survival was 3 months in patients with an NLR > 5 and 7 months in those with an NLR < 5. The NLR might drive therapeutic options in PDAC patients, especially in the setting of metastatic disease.

## 1. Introduction

Pancreatic ductal adenocarcinoma (PDAC) is the 4th leading cause of cancer-related mortality in both men and women [1] and is estimated to become the 2nd within 2030 [2]. PDAC 5-year survival rate is only 6%, as only 20% of patients are eligible for surgery, and chemo/radiotherapy marginally improve survival in advanced diseases [1]. A part of the aggressiveness of PDAC is considered to be due to its complex interactions with the host immune system and fibroblasts. In PDAC, the tumor microenvironment, indeed, stimulates an extensive production of pro-inflammatory cytokines, such as interleukin 2, 6, and 10, and growth factors, such as vascular endothelial growth factor, involved in tumor proliferation and local fibrotic reactions [3,4]. The resultant dense stromal compartment also contributes to the aggressive nature of this tumor [4].

A number of studies have shown that the reciprocal interaction between cancer cells and host immune cells stimulates an extensive systemic inflammation that enhances cancer progression and metastatic spread, by cytokine production and peripheral T lymphocyte suppression, promoting angiogenesis and DNA damage repairing, through growth factor synthesis [5].

In this view, circulating markers associated with inflammation have been explored to determine their association with patients’ outcome in PDAC and other tumor types [6].

Given this importance of the systemic pro-inflammatory response, it is not surprising that changes in the levels of circulating white blood cells (WBC) have been reported in PDAC patients, with an absolute and relative increase of neutrophils and a decrease of lymphocytes. An elevated peripheral blood neutrophil/lymphocyte ratio (NLR) has been extensively investigated as a strong predictor of poor prognosis, negatively affecting both overall and progression free survival, in many different solid tumors such as colorectal cancer, non-small cell lung cancer, renal cell carcinoma, cholangio, and hepatocellular carcinoma [7].

A recent meta-analysis, including 11 prospective studies specifically focusing on PDAC, concluded that elevated NLR at diagnosis is associated to a worse overall survival and surgical outcome and that it negatively affects the response to conventional palliative chemotherapy with gemcitabine or 5-fluoruracile (5-FU), alone or in combination with platinum [8]. The same study also observed that an NLR higher than 5 at diagnosis is associated to undifferentiated and advanced tumors, poor performance status, high C-reactive protein (CRP), and low albumin levels [8].

Other circulating markers of inflammation, such as the platelet–lymphocyte ratio (PLR), alone or in combination with albumin in the Glasgow Modified Prognostic Score (GPS) or the Odonera Prognostic Nutritional Index (PNI), have also been found to be independent variables associated with prognosis in PDAC [9,10,11,12].

However, the prognostic value of these latter variables have been evaluated in few studies, and their significance has been demonstrated mostly in potentially resectable series of PDAC, while their role in patients with advanced disease needs to be extensively investigated.

Finally, only few studies have compared the NLR to these other biomarkers and scores in PDAC patients with conflicting results in terms of prognostic significance of the different scores [13,14].

The aim of this study is therefore to assess the prognostic value of a wide set of circulating biomarkers of inflammatory response and nutrition in PDAC patients.

## 2. Results

### 2.1. Patient General Features and Circulating Markers of Inflammation

Two-hundred and six consecutive patients with PDAC diagnosis were enrolled. A histological or cytological diagnosis was obtained in all cases either from the primary pancreatic lesion or from metastases. Of the 206 patients, 112 (54%) were male, with a median age of 69.9 years (95% CI 68.5–71.4) at diagnosis. Seventy-six (37%) patients had potentially resectable disease at diagnosis; 64 of them underwent surgery with a curative intention (41 with final R0 surgery), 12 were not operated and did not receive active treatment because of advanced age or comorbidities. Forty-six of the 64 operated patients received adjuvant chemotherapy with gemcitabine.

Forty-nine patients (24%) had locally advanced disease. None of them underwent surgery and 35 received chemotherapy with either gemcitabine or 5-FU based regimens. The remaining patients did not receive chemotherapy because of advanced age, comorbidities, or rapid worsening of general conditions.

Eighty-one (39%) patients had distant metastasis at diagnosis. Thirty-nine of them received chemotherapy with either gemcitabine alone, or gemcitabine or 5-FU combos. The remaining 42 patients did not receive an active treatment because of advanced age, comorbidities, or rapid worsening of their general condition.

Data on hemoglobin levels, WBC count, absolute neutrophil and lymphocyte count and platelet count were available for all 206 patients, whereas data on CRP levels were available for 144/206 (69.9%), on total serum proteins and albumin for 189/206 patients, and on Ca 19.9 levels for 173/206 patients. About one-third of patients had an NLR higher than 5 and a CRP/albumin ratio > 1. General features and data on circulating markers of inflammation are summarized in Table 1.

### 2.2. Risk Factor Analysis for Overall Survival

**Figure 1:**
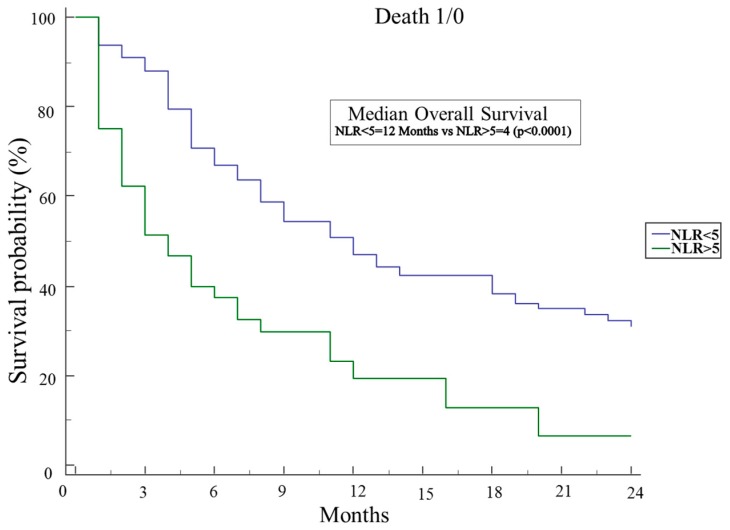
Kaplan–Meier analysis of OS of 206 pancreatic cancer patients with an NLR higher or lower than 5.

At univariate analysis for mortality risk assessment (Table 2), clinical factors significantly associated to worse overall survival (OS) were the age of the patients at diagnosis (HR 1.51 95% CI 1.06–2.14 per increased year; *p* = 0.02), the male sex (HR 1.51 95% CI 1.06–2.14 *p* = 0.02), the maximum diameter of the primary tumor (HR 1.01 95% CI 1.004–1.03 per increased mm; *p* = 0.009), and the presence of distant metastasis at diagnosis (HR 2.97 95% CI 2.06–4.30 *p* < 0.0001). The only factor associated to increased overall survival was access to any chemotherapy during the disease course (HR 0.41 95% CI 0.29–0.59 *p* < 0.0001). As regards the evaluated circulating markers, at univariate analysis, CRP levels (HR 1.04 95% CI 1.001–1.08 per increased unit; *p* = 0.046), an NLR > 5 (HR 2.49 95% CI 1.69–3.67 *p* < 0.0001), an Odonera PNI < 35 (HR 1.57 95% CI 1.05–2.22 *p* = 0.027), and CRP/albumine > 1 (HR 2.02 95% CI 1.26–3.26 *p* = 0.004) were significantly associated with worse OS, whereas GPS and platelet/lymphocyte ratio were not. At multivariate analysis, the maximum diameter of the primary tumor (HR 1.02 per increased mm, 95% CI 1.01–1.03 *p* = 0.046) and the presence of distant metastasis (HR 2.8 95% CI 1.7–4.7 *p* = 0.0001) were confirmed to be significantly and independently associated with decreased OS, as access to any chemotherapy (HR 0.45 95% CI 0.28–0.75 *p* = 0.0022) was confirmed to be associated with increased OS. On the other hand, among the different circulating markers evaluated in our study, only an NLR > 5 (HR 1.9; 95% CI 1.1–3.3 *p* = 0.027) was associated with worse median OS (see Table 2), which was in fact 4 months in subjects with an NLR > 5 and 12 months in those with an NLR < 5 (*p* < 0.0001) (Figure 1).

As elevated NLR was the only circulating marker of inflammatory response significantly associated with OS, we then compared features of the 60 (29.1%) patients with an NLR > 5 to those of the 146 (70.9%) patients with an NLR < 5. Patients with an elevated NLR were older (mean 73.1 years vs. 68.7 *p* = 0.0069) and more frequently male (66.7% vs. 50% *p* = 0.032), and had a higher rate of distant metastasis at diagnosis (61.6% vs. 30.1% *p* < 0.0001). Furthermore, patients with elevated NLR received chemotherapy less frequently (40% vs. 64% *p* < 0.0001).

As previous studies have demonstrated that NLR is associated with survival after surgical resection in PDAC patients, we analyzed the factors associated with OS in the 76 patients with potentially resectable disease at diagnosis separately. At univariate analysis, the mean age of patients (HR 1.07 95% CI 1.04–1.11 *p* < 0.0001) and an NLR > 5 (HR 3.23 95% CI 1.38–7.59 *p* = 0.007) were found to negatively affect OS, whereas hemoglobin levels (HR 0.83 95% CI 0.70–0.98 per g/dL; *p* = 0.03) receiving surgery (HR 0.36 95% CI 0.16–0.83 *p* = 0.03) and access to adjuvant chemotherapy (HR 0.39 95% CI 0.21–0.74 *p* = 0.004) were associated with longer OS. However, at multivariate analysis, only the age of the patients (HR 0.002 95% CI 1.02–1.09 *p* = 0.002) remained a significant factor.

**Figure 2:**
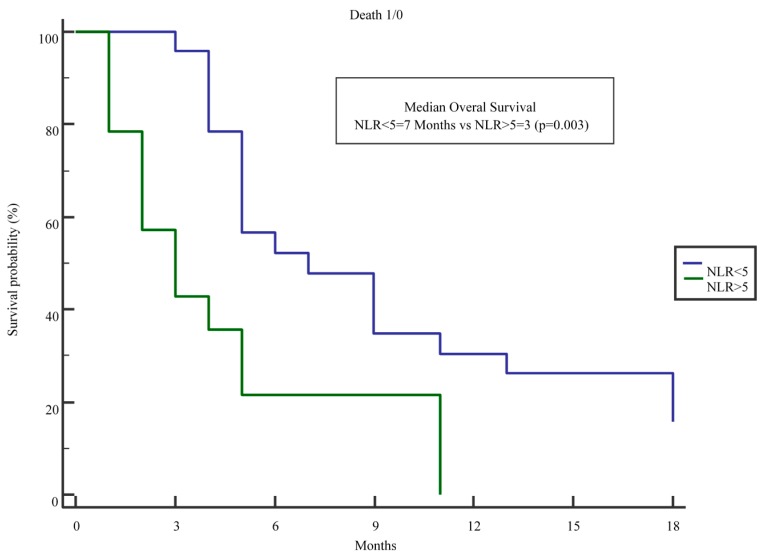
Kaplan–Meier analysis of the OS of 39 Stage IV pancreatic cancer patients receiving standard palliative chemotherapy with gemcitabine- or 5-fluoracile-based regimens, with an NLR higher or lower than 5.

We then evaluated separately the subgroup of 81 patients with distant metastasis at diagnosis. At univariate analysis, the age of the patients (HR 1.03 95% CI 1.003–1.048 per increasing year; *p* = 0.028) and an NLR > 5 (HR 2.05 95% CI 1.21–3.46 *p* = 0007) were associated with worse OS, whereas access to chemotherapy (HR 0.59 95% CI 0.35–0.99 *p* = 0.049) was associated with a better OS. At multivariate analysis, only an NLR > 5 (HR 1.75 95% CI 1.02–3.03 *p* = 0.043) was significantly associated with prognosis (Table 3). Interestingly, when we further restricted the analysis to the 39 subjects with metastatic disease at diagnosis that actually received chemotherapy, those with an NLR > 5 showed a significantly lower OS (Figure 2) when compared to those with an NLR < 5 patients (median 3 vs. 7 months; *p* = 0.003).

As some of the circulating markers and scores were only evaluated in a portion of patients, in order to determine whether this might have caused any bias, we performed the same analyses reported above only in the subgroup of 122 subjects for whom all the parameters were available. In this subgroup, at univariate analysis, the age of the patients at diagnosis (HR 1.03 95% CI 1.002–1.05 per increased year; *p* = 0.03), the maximum diameter of the primary tumor (HR 1.02 95% CI 1.006–1.04 per increased year; *p* = 0.008), and the presence of distant metastasis at diagnosis (HR 2.99 95% CI 1.84–4.87 *p* < 0.0001) were associated with worse OS, and access to any chemotherapy during disease course associated with a better OS (HR 0.37 95% CI 0.23–0.59 *p* < 0.0001). As far as circulating markers, at univariate analysis, an NLR > 5 (HR 2.70 95% CI 1.64–4.45 *p* < 0.0001), CRP/albumine > 1 (HR 2.04 95% CI 1.24–3.35 *p* = 0.005), and platelet/lymphocyte ratio (HR 1.003 95% CI 1.0004–1.005 per increased unit *p* = 0.02) were significantly associated with worse OS, whereas an Odonera PNI (HR 0.95 95% CI 0.90–0.99 per increased unit *p* = 0.02) was associated with a better OS.

## 3. Discussion

The inflammatory response plays an important role in many cancer types including pancreatic cancer. This offers an opportunity to measure simple markers that might be associated with disease outcome in clinical practice. In the past few years, the prognostic role of the neutrophil/lymphocyte ratio has been demonstrated in PDAC patients of different stages [10,11,12,13,15].

Other biomarkers of inflammation and of nutritional status and combined inflammation-based scores have been evaluated in PDAC patients, but the superiority of one to the others in terms of prognostic significance is uncertain [13,14]. Moreover, the majority of studies conducted in this setting were limited to patients with resectable disease who, unfortunately, represent only a small fraction of PDAC patients.

In the present study, the prognostic value of NLR was analyzed in 206 consecutive PDAC patients seen at a single center and compared to that of other inflammation-based prognostic scores such as the Odonera PNI, the Glasgow Prognostic Score, the platelet/lymphocyte ratio and the CRP/albumin ratio. Interestingly, the Ca 19-9 levels were not associated with OS in our analyses, further underlining its limitations and the need of other reliable biomarkers able to predict the outcome of PDAC patients in clinical practice [16].

When we evaluated the whole cohort of PDAC patients, the factors significantly associated with worse OS at the multivariate analysis were age, male sex, tumor size, presence of distant metastasis and, among the above mentioned scores, an NLR > 5, while the access to any chemotherapy was associated with better OS (see Table 2). Patients with an NLR < 5 had a median OS of 12 months compared to 4 months in those with an NLR > 5 (see Figure 1).

Interestingly, patients with elevated NLR were older, had a higher rate of distant metastasis at diagnosis, and received chemotherapy less frequently.

We were, therefore, particularly interested in assessing the prognostic role of NLR in the subgroup of 81 patients with distant metastasis at diagnosis. When we repeated the univariate analysis in this group of patients, the age of the patients and an NLR > 5 were associated with worse OS, whereas access to chemotherapy was associated with a better OS. However, at multivariate analysis, an NLR > 5 was the only variable significantly associated with OS (Table 3).

Kadokura et al. also reported an elevated NLR and a reduced performance status, as only independent variables associated with worse OS in a small group of elderly PDAC patients with advanced disease undergoing chemotherapy [17].

To further assess the possible value of NLR and of the other scores in selecting patients with metastatic disease who might benefit more from chemotherapy, we analyzed the smaller group of 39 subjects with metastatic disease at diagnosis who received chemotherapy. Moreover, in these patients, an NLR > 5 was significantly associated with worse OS (Figure 2), with patients with an NLR > 5 having a disappointing median OS of 3 months compared to a median of 7 months in those with an NLR < 5.

These results are in keeping with findings of poor efficacy of even intensified chemotherapy regimens such as gemcitabine plus Nab-paclitaxel [18] and FOLFOXIRI [19] in patients with advanced PDAC and an elevated NLR before treatment. In another study, however, patients with metastatic PDAC and NLR > 2.5 seemed to benefit more from the combination of gemcitabine with oxaliplatin compared with gemcitabine alone, while this benefit was limited in patients with an NLR < 2.5 [20]. Furthermore, a decrease of NLR after treatment was associated with longer survival.

Given the poor life expectancy of patients with metastatic PDAC and the potential toxicity of chemotherapy, these results would argue against any treatment other than palliation in patients with elevated NLR and poor performance status, while those with elevated NLR but a good performance status might benefit from aggressive combined regimens that might be stopped if NLR does not decrease. Unfortunately, we have not been able to evaluate NLR after chemotherapy to assess the influence of treatment and possibly the relation of NLR changes with treatment efficacy, but this would be an interesting area of future research. It would also be interesting to explore the relation between NLR and treatment response in trials of immunotherapy in PDAC patients or in other tumor types.

The present study confirms previous findings that suggest the prognostic relevance of NLR in PDAC patients and is one of the few to report its superiority as compared with a wide set of other inflammation-based scores [21] in consecutive patients seen in a real-life setting. The results seem particularly important to drive therapeutic choices in the setting of metastatic disease.

The study, however, has several limitations, as it is of retrospective and observational nature and treatments were not standardized. Furthermore, the analysis on the relevance of several markers and scores might have been limited by the fact that some of them could only be evaluated in a portion of the 206 patients, although when we performed the same analyses only in the subgroup of 122 patients for whom all the parameters were available, the results were similar. Moreover, we have not been able to evaluate other serum markers that are associated with the interplay between PDAC cells and the surrounding stroma such as lactate dehydrogenase (LDH). LDH is the enzyme converting pyruvate to lactate in anaerobic conditions, and as PDAC is a poorly vascularized cancer type, hypoxia plays an important role in its progression. Serum LDH levels have been reported to be associated with PDAC prognosis and response to treatment [22,23,24], and the relation between NLR and LDH is another interesting area for future research.

Whether an elevated concentration of neutrophils is only a response to a more aggressive tumor, or it promotes the aggressiveness of the cancer itself is a matter of discussion, and such mechanisms cannot be inferred from the present data. However, findings that circulating tumor cells are surrounded by white blood cells in tumor-adjacent vessels of PDAC patients might support the case for an active role of neutrophils in tumor dissemination [25].

## 4. Patients and Methods

### 4.1. Study Design and Population

A single center cohort study was conducted at the Digestive and Liver Disease Unit of S. Andrea Hospital, University Sapienza of Rome. Consecutive patients with incident, histologically confirmed PDAC, prospectively seen between January 2007 and December 2014 were enrolled.

Patients were interviewed by trained medical doctors who filled in a specific questionnaire to collect data on demographics and symptoms that led to PDAC diagnosis. No proxies were interviewed. All patients gave written informed consent. Data about tumor stage at diagnosis and histological grading were also collected. Patients received appropriate medical treatments and their outcome was recorded in a dedicated database. The study received approval from the local Institutional Review Board (IRB) with protocol number 2013/251 on 27 February 2013.

### 4.2. Measured Biochemical Parameters

We retrospectively collected the following biochemical parameters from patients’ clinical charts: (a) total white blood cell count (expressed as 10^3^ cells/μL), absolute neutrophil and lymphocyte count (expressed as 10^3^ cells/μL), platelet count (expressed as 10,000^3^ cells/μL) hemoglobin value (expressed as g/dL), CRP levels (expressed as mg/dL), total serum proteins and albumin (expressed as g/dL), and Ca 19.9 levels (expressed as UI/mL).

These variables were combined to calculate the following prognostic scores: (1) neutrophil/lymphocyte ratio; (2) Odonera PNI (calculated as 10 × Albumin levels + 0.005 × lymphocyte levels) [12]; (3) Glasgow Prognostic Score (GPS = O was assigned to patients with CRP levels < 1 and albumin levels > 3.5, GPS = 1 to those with CRP > 1 or albumine < 3.5, GPS = 2 to those with CRP > 1 and albumine < 3.5) [12]; (4) platelet/lymphocyte ratio.

Previous findings suggest that, in solid tumors, including pancreatic cancer, an NLR > 5 as well as a CRP/albumin ratio > 1 are associated with worse long-term prognosis, whereas an Odonera PNI score > 40 and a GPS = 0 are favorable prognostic markers [11,12].

For all patients, the results of blood tests that was performed closer to the time of diagnosis was retrospectively selected, but in order to avoid possible bias, whenever that blood test was obtained at the time of a known acute inflammatory process such as sepsis, cholangitis, and pneumonia, the first blood test after recovery from that process was employed.

### 4.3. Statistical Analysis

The above mentioned data were recorded in a dedicated database. A Fisher test for a comparison of proportions for categorical variables and a Student’s *t*-test or Mann–Whitney test for continuous variables were employed. Multiple logistic regression analysis was employed to investigate factors associated with an elevated NLR. Survival probability was calculated with the Kaplan–Meier curve, and Cox analysis was employed to calculate hazard ratios (HR). Tests of statistical significance and confidence intervals were two-sided; *p* < 0.05 was considered to be statistically significant. Dedicated software (MedCalc, Mariakerke, Belgium) was used throughout the study.

## 5. Conclusions

In conclusion, the present study suggests that the NLR is superior to the other inflammation-based prognostic scores and might be employed as an independent predictive marker in patients with pancreatic cancer, with particular clinical usefulness in the setting of patients with metastatic disease who are scheduled to receive chemotherapy.

## Figures and Tables

**Table 1:** General features and circulating inflammatory markers in the 206 evaluated pancreatic cancer patients.

Mean Age (Years)	69.9 (95% CI 68.5–71.4)
Male Sex	112/206 (54.4%)
Disease Stage at diagnosis	
* Potentially Resectable Disease*	76/206 (37%)
* Locally Advanced Disease*	49/206 (24%)
* Distant Metastasis*	81/206 (39%)
Mean primary Tumor Size (mm)	39.4 (95% CI 37.4–41.4)
Patients with Ca 19.9 > 37 (U.I./mL)	141/173 (81.5%)
Patients with an NLR > 5	60/206 (29.1%)
Patients with CRP levels > 1 mg/dL	95/144 (65.9%)
Patients with Albumin levels < 3.5 g/dL	110/189 (58.2%)
Patients with CRP/Albumin Ratio > 1	37/135 (27.4%)
Patients with Odonera PNI score < 35	110/189 (58.2%)
Glasgow Prognostic Score *0*	26/136 (19.1%)
Glasgow Prognostic Score *1*	44/136 (32.3%)
Glasgow Prognostic Score *2*	66/136 (48.5%)

Hb: hemoglobin; Plt: platelets; NLR: neutrophil/lymphocytes ratio; CRP: C-reactive protein; PNI: Prognostic Nutritional Index.

**Table 2:** Factors associated with overall survival (OS) at univariate and multivariate regression analysis.

Patients Features	Univariate Analysis (HR 95% CI)	*p*-Value	Multivariate Analysis (HR 95% CI)	*p*-Value
Age (continuous variable)	1.02 (1.006–1.04)	0.007	1.02 (0.9–1.04)	0.11
Male Sex	1.5 (1.1–2.1)	0.02	1.2 (0.7–1.9)	0.50
Ca 19.9 (>37 U/mL)	0.9 (0.6–1.5)	0.82		
Primary Tumor Size (continuous variable)	1.01 (1.004–1.03)	0.009	1.02 (1.01–1.03)	0.046
Distant metastasis at diagnosis	2.9 (2.1–4.3)	<0.0001	2.8 (1.7–4.7)	0.0001
Access to any Chemotherapy	0.4 (0.3–0.6)	<0.0001	0.45 (0.28–0.75)	0.0022
CRP levels (mg/dL) (continuous variable)	1.04 (1.0009–1.1)	0.046		
NLR (continuous variable)	1.1 (1.01–1.1)	0.014		
NLR > 5	2.5 (1.7–3.7)	<0.0001	1.9 (1.1–3.3)	0.027
Odonera PNI score (continuous variable)	0.96 (0.94–0.99)	0.03		
Odonera < 35	1.6 (1.1–2.2)	0.03		
CRP/Albumin (continuous variable)	1.2 (1.02–1.3)	0.03	1.1 (0.6–1.9)	0.70
CRP/Albumin > 1	2.02 (1.3–3.3)	0.004		
GPS = 0	0.6 (0.4–1.2)	0.17		
GPS = 1	0.8 (0.5–1.3)	0.45		
GPS = 2	1.5 (0.9–2.4)	0.06		
Plt/Lymphocyte Ratio	1.001 (0.9–1.002)	0.35		

CT: chemotherapy; Hb: hemoglobin; Plt: platelets; WBC: white blood cell; NLR: neutrophil/lymphocyte ratio; CRP: C-reactive protein; GPS: Glasgow Prognostic Score.

**Table 3:** Multivariate analysis of risk factors for OS in 81 pancreatic cancer patients with metastatic disease at the time of diagnosis.

Variable	Multivariate (OR 95% CI)	*p*-Value
Mean Age	1.02 (0.99–1.04)	0.09
Access to Chemotherapy	0.73 (0.42–1.26)	0.26
NLR > 5	1.75 (1.02–3.03)	0.04

CT: chemotherapy; NLR: neutrophil/lymphocyte ratio.
